# Supplementary material for: Factors influencing the timely completion of construction projects in Sri Lanka
Source: PLoS One. 2022 Dec 15;17(12):e0278318. doi: 10.1371/journal.pone.0278318 (PMC9754246; doi:10.1371/journal.pone.0278318)
Supplement: S1 Appendix — (DOCX) [file pone.0278318.s001.docx]

# S1 Appendix: Questionnaire

**Factors affecting the timely completion of construction projects in Sri Lanka**

Dear Respondents,

This questionnaire is only for the partial fulfilment of Master of Business Administration degree (MBA) at SLIIT. This research is aimed to analyse the factors affecting the timely completion of construction projects in Sri Lanka. Data will be obtained using a survey questionnaire and please note that this information will be treated in strict confidence and will be used for academic purposes only. No personal information will be published.

Thank You

N.M.De.A. Abeysinghe

**General Information**

(Please mark only one box for the following questions)

1. Are you currently working at a construction project?

Yes [] No []

1. Please select your gender identity

Male [] Female []

1. Please select your age

20-29 years [] 30-39 years [] 40 years and above []

1. Please state your highest educational qualification

Certificate level [] Diploma [] Bachelors degree [] Masters degree [] PhD [] Other []

1. Please state the category of your company/organization

Road construction [] Building construction [] Other []

1. Please state your engagement in the construction sector

Client [] Contractor [] Consultant [] Other []

1. Working experience

Below 5 years [] 5-9 years [] 10-19 years [] 20 years and above []

Please mark the suitable column, considering their significance in delaying the completion of a construction project.

1- Very low significance

2- Low significance

3- Average significance

4- High significance

5- Very high significance

**Section A- Client related factors**

| **Category** |  | **Causes of delay** | **1** | **2** | **3** | **4** | **5** |
| --- | --- | --- | --- | --- | --- | --- | --- |
| Client/Owner related factors | 1 | Changes in design by client during construction |  |  |  |  |  |
|  | 2 | Slowness of the client’s decision-making |  |  |  |  |  |
|  | 3 | Unreasonable project duration given by client |  |  |  |  |  |
|  | 4 | Delay in settling contractor payments by client |  |  |  |  |  |
|  | 5 | Financial difficulties of client |  |  |  |  |  |
|  | 6 | Delay in design approvals |  |  |  |  |  |
|  | 7 | Poor communication with contracting parties |  |  |  |  |  |
|  | 8 | Errors in design and specifications |  |  |  |  |  |

**Section B- Contractor related factors**

| **Category** |  | **Causes of delay** | **1** | **2** | **3** | **4** | **5** |
| --- | --- | --- | --- | --- | --- | --- | --- |
| Contractor related factors | 1 | Poor planning and scheduling |  |  |  |  |  |
|  | 2 | Shortage of skilled subcontractors/suppliers |  |  |  |  |  |
|  | 3 | Financial difficulties of contractors |  |  |  |  |  |
|  | 4 | Disagreements between contractor and other parties |  |  |  |  |  |
|  | 5 | Poor site management, monitoring, and control |  |  |  |  |  |
|  | 6 | Errors during construction |  |  |  |  |  |
|  | 7 | Underestimating the project duration |  |  |  |  |  |
|  | 8 | Regularly changing the subcontractor’s staff |  |  |  |  |  |

**Section C- Consultant related factors**

| **Category** |  | **Causes of delay** | **1** | **2** | **3** | **4** | **5** |
| --- | --- | --- | --- | --- | --- | --- | --- |
| Consultant related factors | 1 | Delay in inspections and completed work approvals |  |  |  |  |  |
|  | 2 | Delay in material and payment approval |  |  |  |  |  |
|  | 3 | Errors in contract documents |  |  |  |  |  |
|  | 4 | Constant design changes by consultant |  |  |  |  |  |
|  | 5 | Delay in preparing and approving drawings and design documents |  |  |  |  |  |
|  | 6 | Lack of experienced consultants |  |  |  |  |  |
|  | 7 | Errors in design documents |  |  |  |  |  |
|  | 8 | Poor coordination and communication |  |  |  |  |  |

**Section D- Resources related factors**

| **Category** |  | **Causes of delay** | **1** | **2** | **3** | **4** | **5** |
| --- | --- | --- | --- | --- | --- | --- | --- |
| Resources related factors | 1 | Shortage of labourers (skilled, semiskilled, unskilled) |  |  |  |  |  |
|  | 2 | Delay of delivering materials to site |  |  |  |  |  |
|  | 3 | Poor material handling at site |  |  |  |  |  |
|  | 4 | Low productivity of labourers |  |  |  |  |  |
|  | 5 | Fluctuation of material prices in the market |  |  |  |  |  |
|  | 6 | Inadequate numbers of equipment |  |  |  |  |  |
|  | 7 | Breakdown of equipment |  |  |  |  |  |
|  | 8 | Personal disagreements between labourers |  |  |  |  |  |

**Section E- External factors**

| **Category** |  | **Causes of delay** | **1** | **2** | **3** | **4** | **5** |
| --- | --- | --- | --- | --- | --- | --- | --- |
| External factors | 1 | Delay in obtaining permissions/approvals from government |  |  |  |  |  |
|  | 2 | Unknown subsurface conditions (soil condition, water table etc.) |  |  |  |  |  |
|  | 3 | Bad weather condition |  |  |  |  |  |
|  | 4 | Accidents during construction |  |  |  |  |  |
|  | 5 | Changes in laws and regulations from the government |  |  |  |  |  |
|  | 6 | Delay in utility services (electricity, water etc.) |  |  |  |  |  |
|  | 7 | Covid-19 pandemic situation |  |  |  |  |  |
