# Supplementary material for: Factors influencing the timely completion of construction projects in Sri Lanka
Source: PLoS One. 2022 Dec 15;17(12):e0278318. doi: 10.1371/journal.pone.0278318 (PMC9754246; doi:10.1371/journal.pone.0278318)
Supplement: Attachment — Submitted filename: Response to reviewer.docx [file pone.0278318.s003.docx]

**Point by point response to reviewer 1**

Dear editor and the reviewer,

We would like to express our profound appreciation to the editor and the reviewers for the valuable comments and suggestions made on our manuscript which were very helpful in revising and improving it.

Please note that line numbers refereed in this document is align with the revised manuscript which has track changes document (attached after the following table).

| **Comments of Reviewer 1** | **Comments of Authors** |
| --- | --- |
| **1. Is the manuscript technically sound, and do the data support the conclusions?**  The manuscript must describe a technically sound piece of scientific research with data that supports the conclusions. Experiments must have been conducted rigorously, with appropriate controls, replication, and sample sizes. The conclusions must be drawn appropriately based on the data presented.  Reviewer #1: **Partly** | Well received & noted. |
| **2. Has the statistical analysis been performed appropriately and rigorously?**  Reviewer #1: **No** | Noted with thank you. |
| **3. Have the authors made all data underlying the findings in their manuscript fully available?**  The [PLOS Data policy](http://www.plosone.org/static/policies.action#sharing) requires authors to make all data underlying the findings described in their manuscript fully available without restriction, with rare exception (please refer to the Data Availability Statement in the manuscript PDF file). The data should be provided as part of the manuscript or its supporting information or deposited to a public repository. For example, in addition to summary statistics, the data points behind means, medians and variance measures should be available. If there are restrictions on publicly sharing data—e.g., participant privacy or use of data from a third party—those must be specified.  Reviewer #1: **Yes** | Sincere appreciate for the feedback and thank you. |
| **4. Is the manuscript presented in an intelligible fashion and written in standard English?**  PLOS ONE does not copyedit accepted manuscripts, so the language in submitted articles must be clear, correct, and unambiguous. Any typographical or grammatical errors should be corrected at revision, so please note any specific errors here.  Reviewer #1: No | Thank you. Comment has been noted and it has been corrected in the revised manuscript. |
| **5. Review Comments to the Author** |  |
| 1. Please underscore the scientific value-added of your paper in your abstract and introduction. | Thank you. Comment has been noted and it has been corrected in the revised manuscript. The scientific value of the study has been added starting from line 41 to 45 in the abstract and also in the introduction section, starting from line 91 to 112, as suggested in the comment.  “The scientific value of the study includes assisting the Sri Lankan construction industry to identify the factors affecting the timely completion of construction projects, and developing mitigation methods and strategies. Also, the stakeholders could properly plan and schedule the construction work by identifying those that need more attention.”  “The objective of this study is to identify the most significant factors that can affect the timely completion of a construction project in Sri Lanka. The scientific value of the study can be elaborated by identifying the difference between the present study and similar studies which have been conducted previously on the Sri Lankan construction industry. This can be explained in four ways. Firstly, the study has analysed the responses given by the main three stakeholders of a construction project, namely, client/owner, contractor, and consultant. Based on their opinions the most influential project delay factors were identified with respect to each type of stakeholder. It is a must to understand perspectives of each stakeholder on construction project delays, and in reality, how each party play the blame game.”  “Secondly, the study was done based on factors related to client/owner, contractor, consultant, resources, and external factors. Based on the results, the most important factor in each group was identified.………..”  “Finally, the study will enable to set up a platform for Civil Engineers in Sri Lanka for knowledge sharing and collaboration with experts ……..” |
| 2. The authors should strengthen their argument with regard to the scientific novelty and also justify the need to develop an integrated assessment framework (Including abstract and introduction). I would like to request the author to emphasize the contributions on practically and academically in the introduction session. | Comment has been well noted. The practical and academical contributions of the paper has been discussed parallelly with the scientific value of the paper, from line 92 to 112 in the Introduction section.  “……..The scientific value of the study can be elaborated by identifying the difference between the present study and similar studies which have been conducted previously on the Sri Lankan construction industry. This can be explained in four ways. Firstly, the study has analysed ……...” |
| 3. There is no literature discussion in the Introduction. What is the literature gap? | Thank you. Comment has been noted. The literature discussion has been added to the introduction section starting from line 116. The research gap has been added from line 227 to 244.  “According to the literature, it can be observed that the focus of many past studies was to analyse the factors affecting the timely completion of construction projects. These studies were sometimes conducted only within a specific type of construction project such as road projects, building projects, infrastructure projects etc. Also, some of the studies analysed either public construction projects or private construction projects and the scope of the study was limited to a certain area in some countries. Therefore, for comprehensiveness, and to fill the research gap, the present study has included every type of construction project and hence, has not limited to projects in certain areas of the country……...” |
| 4. Why use the Relative Importance Index for evaluation? The authors did not conduct adequate literature discussions? | Comment has been noted. The reason for using the Relative Importance Index, has been added from line 235 to 240.  “…….Most of the past studies used the Relative Importance Index (RII) to rank the delay factors, as it was accepted as an accurate method to calculate the importance of each factor. Therefore, RII has been used to rank the project delay factors in the present study,. Furthermore, the delay factors were analysed based on the type of respondents and group of factors. The major group of factors that could affect the timely completion of a construction project was also identified. |
| 5. The author obtained 39 project delay factors without careful literature discussion on project delay factors. This part needs to be explained more carefully | Thank you. Comment has been well noted. Under the Literature Review section, starting from line 138, the significant project delay factors which have been identified in each citation have been mentioned as suggested. |
| 6. How are the results in Table 2 obtained? The author does not specify the content. | Comment has been noted. The calculation of the values included in Table 2, is presented in Sheet 1 in S2 Appendix. This is mentioned in line 316 & 321. Also, the specific equation used to calculate RII is mentioned in line 279.  “The 39 factors were ranked according to their calculated overall relative importance index. The mean value, standard deviation value, calculated RII and the overall rank for each delay factor are shown in Table 2. The calculation of the following values is presented in Sheet 1 in S2 Appendix.”  “The following Equation 1 was used to calculate the RII.  $RII=\frac{\sum_{i=1}^{5} wx}{A\times N}$ (1)  where,  RII = Relative Importance Index  A = 5 (Highest weight)  N = Total count of respondents  w = 1 to 5 (weights given to each factor by each respondent)  x = frequency of responses given to each factor” |
| 7. This section "Qualitative database created from the open-ended question in the questionnaire" is too irrelevant to the full text. How important is this section? Can it be used as a validation result? | Comment has been well noted. The importance of this section has been added starting from line 484 to 495, as it can be used as a validation result for the study.  “……..The importance of obtaining this result was that it depicted a direct relationship with the results obtained from the descriptive analysis of the study, where the most influential group of factors was found to be the “contractor related factors” group. Secondly, the word “inexperience” has been used to describe the lack of skilled labourers, contractors, subcontractors, and suppliers at a construction site. Words such as “materials”, and “insufficient” have also been widely used by the respondents. This can be reconfirmed according to the descriptive analysis, where the “resource related factors” was the second ranked groups of factors. The word “political” has also been used by the respondents, where political influence when selecting contractors, political interference on construction work and the political instability of the country significantly influenced the delays in construction projects. Therefore, this result can be used as a validation for results obtained from descriptive analysis” |
| 8. The conclusion should include following contents such as background, research objective, experiment result, finding and future research and limitations. Moreover, such information would mitigate the excessively theoretical and sophisticated aspect of the paper, as well as confer it applicability and increased relevance to the engineering environment and policymakers. | Comment has been noted. The suggested improvements have been added to the conclusion of the study from line 501 to 511 and from line 542 to 547.  “The main objective of the study was to identify the most critical factors which could affect the timely completion of construction projects in Sri Lanka. The factors were identified through a comprehensive literature review and expert opinion. The developed questionnaire was effectively used to collect data from Civil Engineers in Sri Lanka. The collected data were analysed using the Relative Importance Index. The factors were ranked accordingly to achieve the main objective of the study. The findings of the study could be used by the stakeholders of a construction project to avoid unnecessary delays.  The results of the study would be highly relevant to the engineering environment as well as for the policymakers, as these insights can be used to improve the timely completion of construction projects in Sri Lanka. According to the results of the present study, it should be noted. ……….”  “Even though the study contributes largely to the construction industry in Sri Lanka, there could be some limitations which can be addressed in future research. The sample size could be increased when compared to the large population of Civil Engineers employed in different types of construction work in Sri Lanka. This approach can help for wide coverage and a comprehensive study to gain useful findings to avoid construction delays and related losses.” |
