# Supplementary material for: Factors influencing the timely completion of construction projects in Sri Lanka
Source: PLoS One. 2022 Dec 15;17(12):e0278318. doi: 10.1371/journal.pone.0278318 (PMC9754246; doi:10.1371/journal.pone.0278318)
Supplement: Attachment — Submitted filename: Response to Reviewers.docx [file pone.0278318.s004.docx]

**Point by point response to editor and reviewers**

Dear editor and the reviewers,

We would like to express our profound appreciation to the editor and the reviewers for the valuable comments and suggestions made on our manuscript which were very helpful in revising and improving it.

Please note that the line numbers referred in this document is aligned with the revised manuscript which has track changes.

| **Comments of Reviewer 1** | **Comments of Authors** |
| --- | --- |
| 1. Please rewrite the title by using professional way. | Comment has been well noted and the title is rewritten using professional way. This has included from line 1 to 2 and 26 to 27 as follows,  “A study on timely completion of construction projects in Sri Lanka” |
| 2. Please rewrite the abstract by using professional way. | Comment has been noted and the abstract is rewritten using professional way. This has included from line 29 to 47 as follows,  “Timely completion is a crucial factor for the success of a construction project, especially in the Sri Lankan context. This study aims to identify the most influential factors that affect the timely completion of construction projects in Sri Lanka. Thirty-nine factors were identified through a comprehensive literature review and experts’ opinions. A questionnaire was distributed incorporating the 39 project delay factors, and responses from 163 Civil Engineers were obtained. The Relative Importance Index (RII) analysed and ranked the project delay factors. The top ranked significant project delay factors were identified as shortage of skilled subcontractors/suppliers, shortage of labourers (Skilled, semi-skilled, unskilled), financial difficulties of contractors, delay in delivering materials to the site, and Covid-19 pandemic situation. According to the main three respondent types, i.e., clients/owners, contractors and consultants, the contractor related factors was the key group among others that delay a construction project. The scientific value of the study includes assisting the Sri Lankan construction industry to identify the factors affecting the timely completion of construction projects, and developing mitigation methods and strategies. Also, the stakeholders could duly schedule the construction work by identifying areas that need more attention. The contribution of this study would assist stakeholders to adopt a proactive approach by identifying mistakes on their part and minimising potential issues that lead to construction project delays in Sri Lanka.” |
| 3. Use more technical keywords. | Comment has been noted and it has been corrected in the revised manuscript from line number 49 to 50. |
| 4. The literature review is very shallow with no in-depth review for the previous related work and is not clear; I believe this could be significantly modified to give the important information. | Comment has been well noted and the literature review was modified by providing more information. This has corrected from line 130 to 297. |
| 5. Problem description is not clear; I believe this should be significantly written in a more professional way. Writing style of the manuscript is needed to be improved. | Comment has been well noted. The problem statement is included in the revised manuscript from line 67 to line 93. |
| 6. Check the wiring sequence in the manuscript and improve it. | Comment has been well noted. The writing of the manuscript is improved using professional way. The manuscript was also proofread and edited by a proof-reader. |
| 7. Focus on your novelty and contribution to your paper especially at the conclusion. | Thank you. The comment has been noted. The novelty and contribution of the study is included from line 581 to line 605 as follows,  “The novelty of the study is that the insights would contribute to the engineering environment and are highly relevant to policymakers to improve the timely completion of construction projects in Sri Lanka. According to the present study’s results, the lack of skilled subcontractors and suppliers and the lack of labourers are key issues to be addressed in timely completion of a construction project. Operating with inexperienced and unskilled subcontractors and suppliers may be challenging for the contractors to complete project tasks on time. Further, employing an adequate number of labourers is a must to avoid project delays. The contractors should be funded promptly by the owners so that the former can continue without interruptions to workflow, material shortages etc. As a result, the contractors could support the construction work as scheduled while considering the time and cost constraints. However, this could be much more difficult when the client/owner also faces financial difficulties. When the materials are not available at the construction site for critical activities, it will delay the construction work, whereas the labourers would idle with interruption to work. Therefore, facilitating the transportation of supplies to the construction site without any delays is mandatory. This calls for effective logistics and supplies management. The material and payment processing (for timely approval etc.) should be done efficiently by the consultants to avoid any delays in the construction work. The construction industry suffered a setback amid the initial outbreak of the covid-19 pandemic situation due to lockdowns and work disruptions. Moreover, labourers were scarce (as due to lockdown conditions, they had to be employed in their home areas); practical difficulties were observed when providing accommodation for the labourers (due to fear of Covid-19 health risks, renting was refused instead).” |
| 8. Discuss the formulas in more detail. | Comment has been well noted. The formula which was used in the study was strengthen by adding following paragraph. Please see lines from 356 to line 362.  “Weights were assigned to each factor by each respondent (1= Very low significance, 2= Low significance, 3= Average significance, 4= High significance, 5= Very high significance) were multiplied by the frequency of responses given to each factor, and the total sum of those two values was calculated. The result was divided by the multiplication of the highest weight (5) and the total count of respondents. The ranking of the delay factors was done using the RII. Overall rankings were calculated by combining the responses given by all the respondents.” |
| 9. Use the illustrative flowchart for methodology and sequence. | Comment has been noted and Figure 2 was added to the revised manuscript to show flowchart for methodology and research sequence. This is sated in line number 306 to 307. |
| 10. More focus on questionnaire is needed. | Comment has been well noted. Detail discussion of the questionnaire has been added to the revised manuscript. This has included from line 318 to line 330. |
| 11. Please add all formulas with more details at your manuscript. | Comment has been well noted. The one formula which was used in the study was discussed in more detail from line 347 to line 362. |
| 12. Conclusion should be written in a more professional way. | Comment has been noted and the conclusion is rewritten in a more professional way. This has included from line 570 to 526. |
| 13. Please put list of abbreviations and symbols at the end of the manuscript. | Thank you for the comment. As per the PLOS ONE journal guideline abbreviation should define upon the first appearance in the text. Not as the list of abbreviation. Please check the following link for more details.  <https://journals.plos.org/plosone/s/submission-guidelines#loc-abbreviations>  Authors would like to keep the abbreviation as per the PLOS ONE guidelines. |
| 14. Some editorial and typo errors. | Comment has been noted. The editorial and typo errors were corrected in the revised manuscripts. |
| 15. There are some grammatical errors that need to be fixed. | The revised manuscript was also proofread once the changes were done and therefore, now, we can confirm that the revised manuscript is free of punctuation and grammatical errors. |
| 16. Please add and increase updated References with formatted outline. | Comment has been well noted and the references has been updated in revised manuscript. |

| **Comments of Reviewer 2** | **Comments of Authors** |
| --- | --- |
| 1. The manuscript has been poorly written, while there many flaws. First of all, I have never seen any article which has an introduction section (problem statement) but without any references cited. This should mean that the entire section has been written based on your own findings. | Thank you. Comment has been well noted. The citations were updated in the introduction section, and the problem statement is also included in the revised manuscript from line 67 to line 93. |
| 2. Literature search has many missing articles on the subject and I don't understand the rationale behind the separation of the studies according to different countries, as this study is not a review study. | Comment has been noted. The reason for separating the studies based on the continents was included from line 133 to line 138. |
| 3. Literature review section is too long but not comprehensive. | Thank you. Comment has been well noted and the literature review was modified by providing important information in the revised manuscript from line number 130 to 297. |
