# Supplementary material for: Factors influencing the timely completion of construction projects in Sri Lanka
Source: PLoS One. 2022 Dec 15;17(12):e0278318. doi: 10.1371/journal.pone.0278318 (PMC9754246; doi:10.1371/journal.pone.0278318)
Supplement: Attachment — Submitted filename: REVIEW REPORT.docx [file pone.0278318.s005.docx]

# REVIEW REPORT/COMMNETS

# TITLE: A study on timely completion of construction projects in Sri Lanka

**Suggested improvement in Title**: Factors influencing the timely completion of construction projects in Sri Lanka

**ABSTRACT**

1. Sampling technique used was not captured in the abstract.

**KEYWORDS-improve the keywords, see suggestions**

1. Construction projects, Time overrun, Relative Importance Index (RII), Delay factors,
   Sri Lanka

**INTRODUCTION SECTION**

1. There a lot of grammar errors and poor sentence construction. Avoid short sentences that do not make a complete sense. There a lot of these type of sentences, for example,
2. The introduction section says a lot about your work. You have to strengthen the research problem and gap. Consult more literature, there a lot of them on time performances factors in construction. Just 7 citations in this very important subject are not enough. Remove less important sentences; be concise in your approach.

**LITERATURE REVIEW SECTION**

1. The review section is satisfactory, however, some grammar editing is required

**METHODOLOGY SECTION**

**Overall, the methodology adopted is satisfactory, bit it can be made better by attending to the followings;**

1. Why only civil engineers: Justify please. Could you not have obtained robust data if other experts in the construction industry were included?
2. Sampling method adopted in the electronic administration of questionnaire was not stated.
3. Justify the choice of questionnaire. Why was questionnaire used in this study?

**DATA ANALYSIS SECTION**

1. There was the lack of a clear explanation/description of the position of the outcome of this study with existing knowledge in the same subject area. Relate your results/finding to existing reports on the subject area. Please discussion your findings. How does it relate to the review you did?

**CONCLUSIONS AND RECOMMENDATIONS**

1. Please review this section to showcase and improve on the efforts put in previous sections. Especially regarding key factors and relationships. Avoid unnecessary ingredients in the conclusion section.
2. Does this study have limitations? What recommendation do you have for future studies?
3. Does this study have implications?

**REFERENCES**

Revisit the reference list and make sure it conforms to the journals’ guide/format
