# Supplementary material for: Factors influencing the timely completion of construction projects in Sri Lanka
Source: PLoS One. 2022 Dec 15;17(12):e0278318. doi: 10.1371/journal.pone.0278318 (PMC9754246; doi:10.1371/journal.pone.0278318)
Supplement: Attachment — Submitted filename: Response to Reviewers.docx [file pone.0278318.s007.docx]

**Point by point response to editor and reviewers**

Dear editor and the reviewers,

We would like to express our profound appreciation to the editor and the reviewers for the valuable comments and suggestions made on our manuscript which were very helpful in revising and improving it.

Please note that the line numbers referred in this document is aligned with the revised manuscript which has track changes.

| **Comments of Editor** | **Comments of Authors** |
| --- | --- |
| **Journal Requirements:** Please review your reference list to ensure that it is complete and correct. If you have cited papers that have been retracted, please include the rationale for doing so in the manuscript text, or remove these references and replace them with relevant current references. Any changes to the reference list should be mentioned in the rebuttal letter that accompanies your revised manuscript. If you need to cite a retracted article, indicate the article’s retracted status in the References list and also include a citation and full reference for the retraction notice. | Thank you very much and comment has been well noted. Reference list has been created as per the PLOS ONE journal guidelines. PLOS ONE uses “Vancouver” style and authors also double check the referencing style with the recently published articles in PLOS ONE. Authors would like to confirm reference list is complete and correct. As per the reviewer 1 feedback below, following two references have been added to the introduction section of the revised manuscript to strengthen the research problem and gap of the study.  3. Singh R. Delays and cost overruns in infrastructure projects: extent, causes and remedies. Economic and Political Weekly. 2010:43-54.  4. Catalão FP, Cruz CO, Sarmento JM. The determinants of time overruns in Portuguese public projects. Journal of Infrastructure Systems. 2021;27(2):05021002.  In our previous manuscript there were 41 references and it has increased to 43 with the current revised manuscript. Since this study is related to the Sri Lanka authors would like to keep the Sri Lankan literature. |
| **Addition Editor Comments:** |  |
| **Comments of Reviewer** | **Comments of Authors** |
| 1. Suggested improvement in Title: Factors influencing the timely completion of construction projects in Sri Lanka | Thank you. Comment has been well noted and the suggested title has been used for as the new title of the revised manuscript from line 2 to 3 and 28 to 29.  “Factors influencing the timely completion of construction projects in Sri Lanka” |
| 2. Sampling technique used was not captured in the abstract. | Comment has been well noted. The sampling technique which was used in the has been included in the revised manuscript line number 36 to 37.  “A questionnaire incorporating the 39 project delay factors was distributed among 163 Civil Engineers, and responses were obtained. Random sampling method was adopted to select the sample.” |
| 3. Improve the keywords, see suggestions; Construction projects, Time overrun, Relative Importance Index (RII), Delay factors, Sri Lanka | Comment has been well noted. The suggested keywords have been added to the revised manuscript line number from 51 to 52.  **“Keywords:** Construction projects, Time Overrun, Relative Importance Index (RII), Delay factors, Sri Lanka” |
| 4. There are a lot of grammar errors and poor sentence construction. Avoid short sentences that do not make a complete sense. | Comment has been well noted. Grammar errors and poor sentence construction have been resolved in the revised manuscript. |
| 5. The introduction section says a lot about your work. You have to strengthen the research problem and gap. Consult more literature, there a lot of them on time performances factors in construction. Just 7 citations in this very important subject are not enough. Remove less important sentences; be concise in your approach. | Comment has been well noted. More literature have been added in the revised manuscript from line 68 to 84. The following references have been added to revised manuscript to strengthen the research problem and the research gap.  3. Singh R. Delays and cost overruns in infrastructure projects: extent, causes and remedies. Economic and Political Weekly. 2010:43-54.  4. Catalão FP, Cruz CO, Sarmento JM. The determinants of time overruns in Portuguese public projects. Journal of Infrastructure Systems. 2021;27(2):05021002.  “The nature of the construction industry can be considered as uncertain. Construction projects differ from each other depending on the project size, project objectives, project duration, etc. Every project is unique on its own and no project has the same characteristics. Even though the construction projects nowadays use advanced and new project management theories and technologies, the delay in the completion of projects cannot be mitigated [3].  The time deviation of a construction project can be defined as the difference between specified project duration and the real project duration. There can be three types of time deviations in a construction project. Firstly, is a negative deviation, where the real duration is less than the specified duration. Secondly, there is the no particular deviation type, where the specified duration and the real duration are the same. Thirdly, is the positive deviation, where the real duration is greater than the specified duration. This positive deviation is also known as the time overrun, where the delays in the project completions occur. When the delay period is long, consequently the effects will also be greater/significant, which can exert a negative impact on the project. For the successful completion of a project, cost, quality as well as time, should be properly utilised [4].” |
| 6. The review section is satisfactory. However, some grammar editing is required. | Comment has been well noted. Grammar editing has been done for the revised manuscript. |
| 7. Why only civil engineers: Justify please. Could you not have obtained robust data if other experts in the construction industry were included? | Comment has been well noted. The reason for obtaining data from Civil Engineer has been included in the revised manuscript line number from 323 to 325.  “….Qualified Civil Engineers were selected as the population, since they are engaged in the construction work under all three stakeholder groups namely, client/owner, consultant and contractor…..” |
| 8. Sampling method adopted in the electronic administration of questionnaire was not stated. | Comment has been well noted. The sampling method is included in line 328 in the revised manuscript.  “…Then, the final questionnaire was distributed to 1,416 respondents selected using a random sampling method from 2,716 Civil Engineers included in the list…..” |
| 9. Justify the choice of questionnaire. Why was questionnaire used in this study? | Comment has been well noted. The reason for selecting questionnaire method has been include from line 302 to 304 in the revised manuscript.  “As the research strategy, survey strategy was adopted for the study where a questionnaire was developed in which the project delay factors were included. The respondents were able to rank each factor according to their significance, with the use of a questionnaire….” |
| 10. There was the lack of a clear explanation/description of the position of the outcome of this study with existing knowledge in the same subject area. Relate your results/finding to existing reports on the subject area. Please discussion your findings. How does it relate to the review you did? | Comment has been well noted. This has been included in the revised manuscript from line 389 to 400.  “…This result alligned with the results obtained by Yap, Goay (11) and Wang, Ford (22). Shortage of labourers (Skilled, semi-skilled, unskilled) was the second most significant factor (RII=0.8245) categorised under resource-related factors. This result can be further confirmed by the results obtained by Durdyev, Omarov (6) who conducted a study in Cambodia to identify the time overrun factors in residential building construction projects in the country. These factors seem realistic in the construction industry as most subcontracts/suppliers and labourers are not skilled. The third highest ranked factor was the financial difficulties of contractors (RII=0.8233), another contractor-related factor. In Malaysia, two research studies (9), (11) confirmed that the financial difficulties of the contractor play a major role in delaying construction projects, while Prasad, Vasugi (23) have also received the same result in the research they conducted in India…..” |
| 11. Please review this section to showcase and improve on the efforts put in previous sections. Especially regarding key factors and relationships. Avoid unnecessary ingredients in the conclusion section. | Comment has been well noted. This has been included in the revised manuscript from line 561 to 584.  “…..These factors were categorised into five groups namely, client/owner related factors, contractor related factors, consultant related factors, resource related factors and external factors. A questionnaire was developed incorporating questions relevant to these factors, which was effective in collecting data from the selected 163 Civil Engineers in Sri Lanka. The collected data were analysed using the RII. The factors were ranked accordingly to achieve the main objective of the study. The top ten project delaying factors were identified as, “Shortage of skilled subcontractors/suppliers”, “Shortage of labourers (Skilled, semi-skilled, unskilled)”, “Financial difficulties of contractors”, “Delay of delivering materials to site”, “Covid-19 pandemic situation”, “Fluctuation of material prices in the market”, “Poor planning and scheduling”, “Inadequate numbers of equipment”, “Poor site management, monitoring, and control” and “Delay in obtaining permissions/approvals from government”. According to the clients/owners and consultants, “Financial difficulties of contractors” was the most influential factor which delays a construction project. The contractors claimed that the “Shortage of skilled subcontractors/suppliers” was the most significant project delaying factor. Also, the most important group of factors was identified as the contractor related factors group. This was further confirmed by the qualitative database obtained by the respondents’ opinions. The most influential factor under the client/owner related factors was identified as the “Financial difficulties of clients”. “Shortage of skilled subcontractors/suppliers” ranked at the top of contractor related factors. “Delay in material and payment approval” and “Shortage of labours (Skilled, semi-skilled, unskilled)” were the most significant factors under consultant related factors and resource related factors respectively. When it comes to the external factors, “Covid-19 pandemic situation” was identified as the top ranked project delaying factor…..” |
| 12. Does this study have limitations? What recommendation do you have for future studies? | Comment has been well noted. Limitations of the study has been included in the revised manuscript from line 620 to 626. Recommendations has been included from line 628 to 643.  “Even though the study contributes largely to the construction industry in Sri Lanka, there could be some limitations which can be addressed in future research. The sample size could be expanded to be representative, compared to the large population of Civil Engineers employed in different types of construction work in Sri Lanka. This approach can assist for wide coverage and a comprehensive study to gain useful findings to avoid construction delays and related losses.”  “Based on the study's results, the financial difficulties of both contractors and clients should be evaluated for the successful completion of construction projects. Also, experienced subcontractors, suppliers and labourers should be available to avoid project delays. A skilled workforce is a must but lacking in the Sri Lankan setting. A transparent approach and unbiased selection of suitable contractors are mandatory to minimise root causes.  The timely completion of any construction project has a significant effect on the success of the project. Currently, the Sri Lankan construction industry is undergoing a booming phase, meaning meeting high demand and avoiding interruptions are vital. Therefore, the construction projects must be delivered on time without any delays, as it can also affect the country's overall economy. Therefore, the results of this study will be useful for the stakeholders to identify areas that deserve much focus and attention. Based on the findings of this research, the stakeholders can plan adequately, schedule, control and monitor the construction activities. Further, they can avoid losing revenue, undue delays, incurring additional costs and ensure credibility. The Sri Lankan government can use the findings of this study to foresee the reasons for delays in various construction projects and devise necessary mitigation measures.” |
| 13. Does this study have implications? | Comment has been well noted. Policy implications have been added in the revised manuscript from line 629 to 643.  “Based on the study's results, the financial difficulties of both contractors and clients should be evaluated for the successful completion of construction projects. Also, experienced subcontractors, suppliers and labourers should be available to avoid project delays. A skilled workforce is a must but lacking in the Sri Lankan setting. A transparent approach and unbiased selection of suitable contractors are mandatory to minimise root causes.  The timely completion of any construction project has a significant effect on the success of the project. Currently, the Sri Lankan construction industry is undergoing a booming phase, meaning meeting high demand and avoiding interruptions are vital. Therefore, the construction projects must be delivered on time without any delays, as it can also affect the country's overall economy. Therefore, the results of this study will be useful for the stakeholders to identify areas that deserve much focus and attention. Based on the findings of this research, the stakeholders can plan adequately, schedule, control and monitor the construction activities. Further, they can avoid losing revenue, undue delays, incurring additional costs and ensure credibility. The Sri Lankan government can use the findings of this study to foresee the reasons for delays in various construction projects and devise necessary mitigation measures.” |
| 14. Revisit the reference list and make sure it conforms to the journals’ guide/format | Comment has been well noted. Reference list has been created as per the PLOS ONE journal guidelines. Please check the following link for more details. <https://journals.plos.org/plosone/s/submission-guidelines/#loc-reference-style> |
